# Hippocampal Glycerol-3-Phosphate Acyltransferases 4 and BDNF in the Progress of Obesity-Induced Depression

**DOI:** 10.3389/fendo.2021.667773

**Published:** 2021-05-13

**Authors:** Yin-qiong Huang, Yaofeng Wang, Keyue Hu, Shu Lin, Xia-hong Lin

**Affiliations:** ^1^ Department of Endocrinology, The Second Affiliated Hospital of Fujian Medical University, Quanzhou, China; ^2^ Centre of Neurological and Metabolic Research, The Second Affiliated Hospital of Fujian Medical University, Quanzhou, China; ^3^ Diabetes and Metabolism Division, Garvan Institute of Medical Research, 384 Victoria Street, Darlinghurst, Sydney, NSW, Australia; ^4^ Department of Endocrinology, The Seventh Affiliated Hospital of Sun Yat-sen University, Shenzhen, China

**Keywords:** glycerol-3-phosphate acyltransferases 4, depression, high fat diet, hippocampus, ventromedical hypothalamus, inflammation

## Abstract

**Background:**

Obesity has been reported to lead to increased incidence of depression. Glycerol-3-phosphate acyltransferases 4 (GPAT4) is involved in triacylglycerol synthesis and plays an important role in the occurrence of obesity. GPAT4 is the only one of GPAT family expressed in the brain. The aim of this study is to investigate if central GPAT4 is associated with obesity-related depression and its underlying mechanism.

**Results:**

A high-fat diet resulted in increased body weight and blood lipid. HFD induced depression like behavior in the force swimming test, tail suspension test and sucrose preference test. HFD significantly up-regulated the expression of GPAT4 in hippocampus, IL-1β, IL-6, TNF-α and NF-κB, accompanied with down-regulation of BDNF expression in hippocampus and ventromedical hypothalamus, which was attributed to AMP-activated protein kinase (AMPK) and cAMP-response element binding protein (CREB).

**Conclusion:**

Our findings suggest that hippocampal GPAT4 may participate in HFD induced depression through AMPK/CREB/BDNF pathway, which provides insights into a clinical target for obesity-associated depression intervention.

## Introduction

Obesity is a metabolic disorder caused by excessive accumulation of fat due to increased energy intake. Both environmental factors and genetic factors are responsible for the development of obesity (1, 2). With the development of social economy and lifestyle changes, the incidence of obesity is increasing over years, and it has become a serious public health problem. The main clinical consequences of obesity include diabetes, cardiovascular disease, respiratory distress syndrome, sleep disorders, asthma, and tumors, as well as various mental and psychological diseases (3, 4).

Both epidemiological and clinical studies have shown that there is a positive correlation between obesity and depression. Obese people have a significantly increased risk of depression (5, 6). Body mass index (BMI) is positively correlated with the degree of clinical depressive symptoms (7). However, although high-energy food can alleviate negative emotions and bad moods in a short period of time, the weight gain caused by long-term consumption will aggravate the depressive symptoms in patients (8, 9). Some scholars have also discovered that depression and obesity have the same candidate genes from the perspective of genetics. Depression and obesity have a high incidence of comorbidities, which seriously endanger the health of patients, but the mechanism is still unclear.

Obesity is related to a high-risk of depression. Both clinical studies and animal experiments have showed that there is a positive relationship between the two. However, the neuropathophysiological mechanism of depression caused by obesity remains unclear. Based on previous studies, neuroinflammation have been implicated in the development of depression. Inflammation, especially neuroinflammation, is an important link between obesity and depression. A high-fat diet activates the inflammatory response in the animal’s brain. Animal experiments have shown that IL-1β in the brain can mediate chronic stress-induced depression-like behaviors, while IL-1β receptor knockout mice will not show depression-like behaviors after stress (10).

Glycerol-3-Phosphate Acyltransferases 4 (GPAT4) is the key rate-limiting enzyme in the synthesis of triacylglycerols in the glycerophosphate pathway. Gene overexpression and gene knockout experiments (11, 12) confirmed that GPAT4 plays an important role in the development of obesity, liver steatosis and insulin resistance. Compared with wild-type mice, GPAT4-/- (gene knockout) mice lose weight, have subcutaneous lipodystrophy, reduce triacylglycerol (TAG) content in adipose tissue and liver, and improve insulin resistance (13). And a recent study (14) found that overexpression of GPAT4 in the liver of mice resulted in liver insulin resistance and thus impairs liver glucose metabolism, leading to increased liver gluconeogenesis and reduced glycogen synthesis, and ultimately destroys glucose homeostasis, indicating that GPAT4 may be a new drug target for potential prevention and treatment of obesity, insulin resistance and type 2 diabetes (15). GPAT4 is important in the development of obesity. Besides, GPAT4 is the only one in the GPAT family that is expressed in the brain (16, 17). However, whether central GPAT4 is involved in the development of depression remains unclear.

Brain-derived neurotrophic factor (BDNF) is mainly expressed in the central nervous system, especially in hippocampus and cortex. BDNF has been found to regulate food intake and energy metabolism in the central nervous system, promote body movement, suppress appetite, and improve the leptin resistance and insulin resistance (18). Besides, BDNF is also the main regulator of synaptic plasticity and memory formation (19). It is the major regulator of maintaining neuronal function, regeneration and repair, and preventing neuronal degeneration (20). Obese mice experienced a decline in cognitive function after a long-term high-fat diet, accompanied by corresponding pathological changes in hippocampal neurons, which are closely related to the decline of BDNF levels in hippocampus. Recent studies have found that obesity can induce hippocampal inflammation and impairs emotion related to the hippocampus (21).

High-fat-induced obese mice can lead to the inflammatory state in hippocampus of the mouse and the decrease of BDNF. Studies showed that GPAT4 is also highly expressed in the hippocampus, and GPAT4 is the key rate-limiting enzyme for the synthesis of triacylglycerols in the glycerophosphate pathway. Therefore, this study aims to investigate whether central GPAT4 is associated with obesity-related depression and its underlying mechanism.

## Materials and Methods

### Laboratory Animals and Reagents

Twenty-four 5-week-old male C57BL/6 mice were purchased from the Animal Experimental Center of Fujian Medical University. All mice were housed under standard conditions (constant temperature, constant humidity conditions, and a 12-h light/dark cycle), with free access to food and water. The study followed the National Guidelines for Laboratory Animal Welfare and was approved by the Experimental Animal Ethics Committee of the Second Affiliated Hospital of Fujian Medical University (2020-388).

### Establishment of Mice Model

The twenty-four mice were acclimatized for 1 week before conducting the experiments. Then, they were randomized to two groups: normal diet group (ND group, n = 12) and high fat diet group (HFD group, n = 12). The ND group was fed a normal diet (Research Diets, D12450h), and the HFD group were fed a high-fat diet (Research Diets, D12451) for 8 weeks. Six mice in each group were subjected to brain tissue isolation for subsequent qPCR, and the remaining six mice were subjected to perfusion to make frozen sections of brain tissue for *in situ* hybridization.

### Behavior Tests

#### Forced Swimming Test (FST)

The mice were forced to swim for 6 min in a transparent cylindrical container (40cm in height and 20cm in diameter) containing clean water (24°C, 20cm in depth). The duration of immobility state was measured (22).

#### Tail Suspension Test (TST)

In brief, the mice were suspended approximately 28 ± 2 cm off the floor by fixing its tail (2 cm from the tip of the tail) on the hook. During the experiment immobility time of the mice were automatically recorded for 6 mins (22, 23).

#### Sucrose Preference

Before starting the experiment, the mice were singly housed and trained to freely drink water and sucrose water in two bottles (the positions of the two water bottles were switched every day) and the daily intake of sucrose water and regular water was recorded for 1 week. The experiment was started after a stable sucrose water consumption was evident. After 21 days, the sucrose preference test was performed. The detailed test protocol was as follows: after a 12 h period of water fasting, the animals were allowed free access to two bottles respectively containing water and 1% sucrose solution. This test lasted for two hours. Sucrose preference was calculated as the ratio of sucrose water intake to the total volume of liquid intake (24, 25).

#### Lipid Measurement

Fasting lipid levels including total cholesterol, low density lipoprotein and triglyceride were measured with an automatic biochemistry analyzer.

#### *In Situ* Hybridization (ISH) by RNAscope Technology to Determine the mRNA Expression of BDNF in the Hippocampus and Ventromedical Hypothalamus

The animals were perfused with phosphate buffered solution (PBS) at a pH of 7.4 by a cannula inserted in the left ventricle after anesthesia, followed by 4% paraformaldehyde. After perfusion, the brains were immediately removed and were fixed in 4% paraformaldehyde in PBS at 4°C for 12 h and passed through 20 and 30% sucrose gradients prior to embedding in optimum cutting temperature compound (OCT). 20μm tissue sections were air-dried at −20°C and moved to −80°C for long-term storage. Commercially available RNAscope brown reagent kit and RNAscope probes (Advanced Cell Diagnostics, Hayward, CA, Cat No. 322300) were used for transcript detection. ISH was performed according to the manufacturer’s instructions for fixed-frozen tissue. The detection was operated in a hybridization oven (HybEZ™, ACD) with RNAscope Probe- Mm-BDNF (ACD 518751). Each set of probes contained a tag that enabled target transcription to be visualized in a brown color. To compare the expression differences between the two groups, we quantified the integral optical density (IOD) of positive BDNF staining using ImageJ and normalized it by stained area. The mean intensities from three random areas of the same size in target areas were measured for each probe.

#### Quantitative RT-PCR (qRT-PCR)

At the end of each experiment, a microdissection procedure was used to isolate hippocampus. Total RNA was extracted with TRIzol (RNAiso Plus) method (Takara, Japan). RNA was reversed transcribed into cDNA using the two-step method with PrimeScript™ RT reagent Kit with gDNA Eraser (Takara, Japan), according to the manufacturer’s instructions. mRNA qRT-PCR was performed with the TB Green™ Premix Ex Taq™ (TliRNaseH Plus) (Takara, Japan) according to the manufacturer’s instruction. The procedure was 95°C for 1 min; 95°C for 15 s and 60°C for 34 s, for 40 cycles; 95°C for 15 s, 60°C for 1 min and 95°C for 15 s. The primers were shown in (**Table 1**).

**Table 1:** Primers of qRT-PCR.

Gene		Primers sequence
β-actin	Forward	5’ CTACCTCATGAAGATCCTGACC 3’
	Reverse	5’ CACAGCTTCTCTTTGATGTCAC 3’
GPAT4	Forward	5’ AACCTCCTGGGTATCTCCCTG3’
	Reverse	5’ CCGTTGGTGTAGGGCTTGT3’
IL-1β	Forward	5’ GAAATGCCACCTTTTGACAGTG3’
	Reverse	5’ TGGATGCTCTCATCAGGACAG3’
IL-6	Forward	5’ CTCCCAACAGACCTGTCTATAC 3’
	Reverse	5’ CCATTGCACAACTCTTTTCTCA 3’
TNF-α	Forward	5’ ATGTCTCAGCCTCTTCTCATTC 3’
	Reverse	5’ GCTTGTCACTCGAATTTTGAGA 3’
NF-кB	Forward	5’ CAAAGACAAAGAGGAAGTGCAA 3’
	Reverse	5’ ACTTGATGATCCTCGAGATGTC 3’
AMPK	Forward	5’ GTCAAAGCCGACCCAATGATA3’
	Reverse	5’ CGTACACGCAAATAATAGGGGTT3’
CREB	Forward	5’ AGCAGCTCATGCAACATCATC3’
	Reverse	5’ AGTCCTTACAGGAAGACTGAACT3’

β-actin was used as mRNA reference gene, with the 2−ΔΔCt method used for quantitation. Triplicate experiments were performed and repeated at least 3 times.

### Statistical Analyses

All statistical analyses were performed using the SPSS Statistics 20 software. Data have been expressed in terms of mean ± standard deviation. Statistical significances between two groups of data were determined using unpaired, two-tailed Student’s *t*-test. A *P* value >0.05 was not considered significant, *P* value <0.05 was labeled as (*), *P* value <0.01 was labeled as (**), *P* value <0.001 was labeled as (***).

## Results

### Metabolic Phenotype in Dietary-Induced Obesity Mice

**Figure 1:**
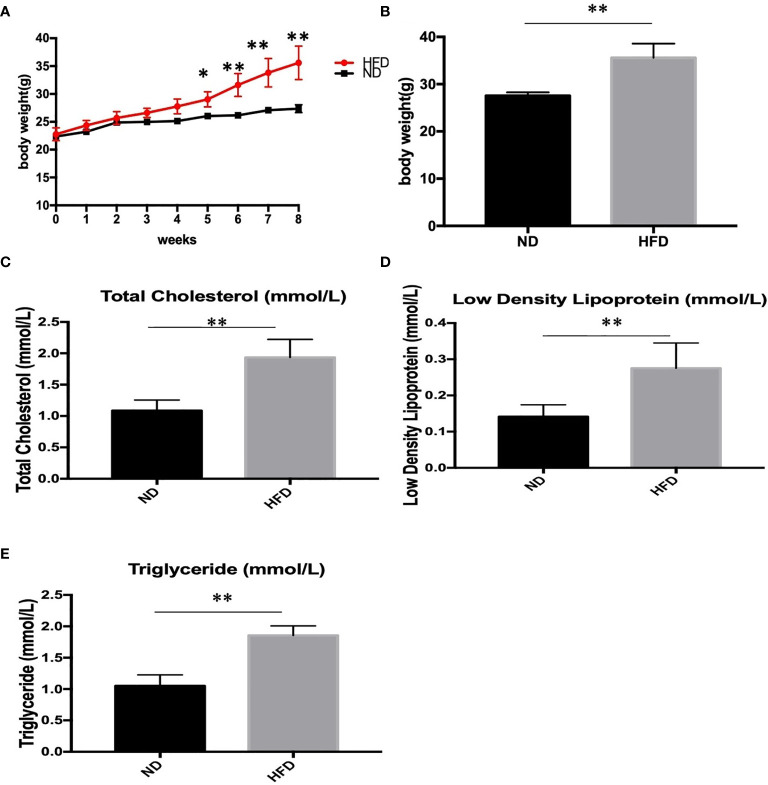
Body weight and blood lipid level after 8 weeks of HFD and ND. **(A)** body weight changes during the 8 weeks; **(B)** Body weight after 8 weeks; **(C)** total cholesterol; **(D)** low density lipoprotein; **(E)** triglyceride. Results are mean ± standard deviation, *P value < 0.05, **P value < 0.01, comparison between the mice fed HFD and ND. (n=6).

To determine the metabolic phenotype of mice after a fat-dense diet, we measure the body weight of mice every week. Besides, at the end the of experiment, blood lipid level was measured. We found that 8 weeks of high-fat diet caused increased body weight (**Figures 1A, B**), and blood lipid level including total cholesterol (**Figure 1C**), low density lipoprotein(**Figure 1D**), and triglyceride (**Figure 1E**), compared with ND group.

### Depression-Like Phenotype in Dietary-Induced Obesity Mice

**Figure 2:**
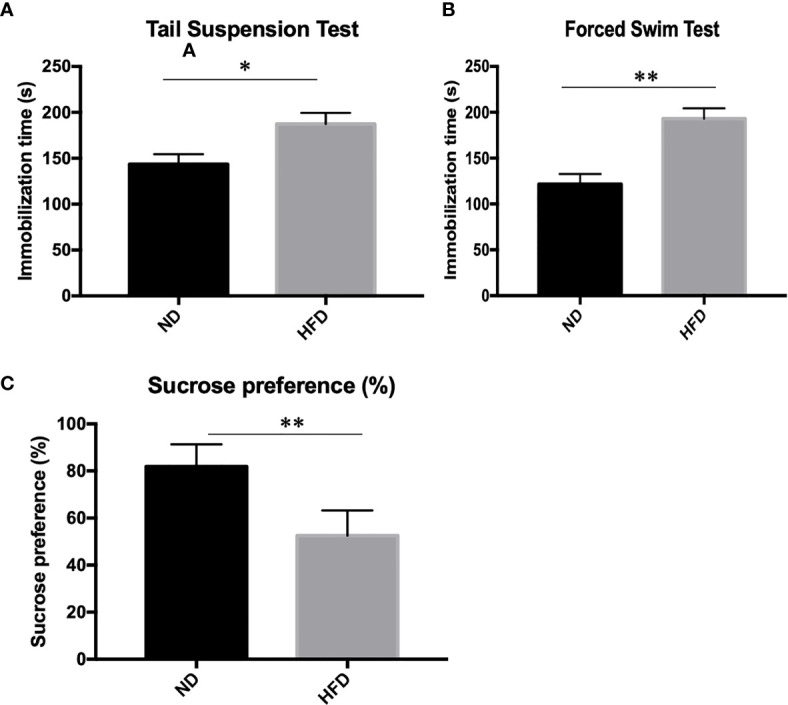
Depression-like phenotype in dietary-induced obesity mice. **(A)** forced swim tests; **(B)** tail suspension tests; **(C)** Sucrose preference test. *P value < 0.05, **P value < 0.01, comparison between the mice fed HFD and ND. (n=6).

To determine whether the consumption of a fat-dense diet plays a causative role in the development of depression, we examined depression-related behaviors among mice fed a ND or HFD. After 8 weeks of high-fat diet (HFD), we examined depression-related behaviors including forced swimming test, tail suspension test and sucrose preference test. Increased immobilization time was observed during forced swim tests (**Figure 2A**) and tail suspension tests (**Figure 2B**) in HFD mice. Also, consumption of sucrose solution (**Figure 2C**) was less in HFD mice compared with control mice on a normal diet.

### GPAT4 Expression in Hippocampus Was Decreased After High Fat Diet

**Figure 3:**
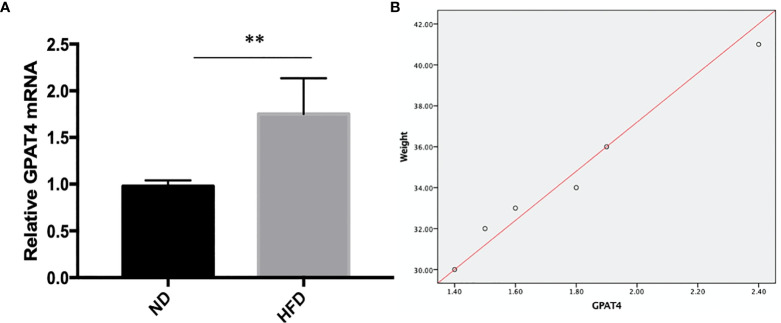
**(A)** GPAT4 expression in hippocampus was decreased after high fat diet. **P value < 0.01, comparison between the mice fed HFD and ND.**(B)** Correlation between GPAT4 expression and body weight. (n=6).

GPAT4 is the key rate-limiting enzyme in the synthesis of triacylglycerols in the glycerophosphate pathway and plays an important role in the development of obesity. Besides, GPAT4 is the only one in the GPAT family that is expressed in the brain. To investigate whether central GPAT4 is involved in the development of obesity-related depression, GPAT4 mRNA expression in hippocampus was measured after 8 weeks of high-fat diet (HFD). We found that compared with ND group, GPAT4 mRNA expression in hippocampus was significantly up-regulated in HFD group (**Figure 3A**). We further investigated the correlation between GPAT4 and body weight, we found that the GPAT4 was positive correlated with body weight (p<0.05) (**Figure 3B**)

### High Fat Diet Induced Inflammation in Hippocampus

**Figure 4:**
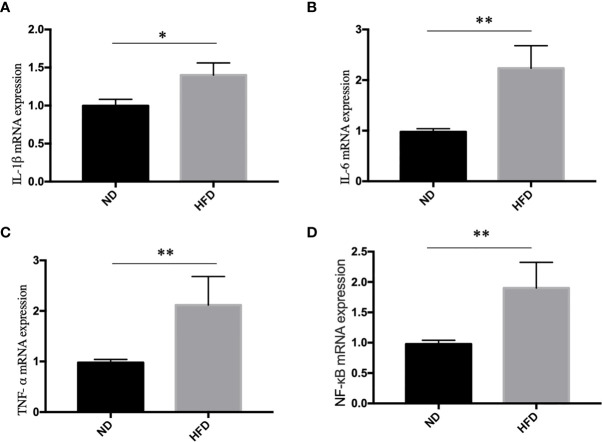
High fat diet induced inflammation in hippocampus. **(A)** IL-1β; **(B)** IL-6; **(C)** TNF- α; **(D)** NF-κB. *P value < 0.05, **P value < 0.01, comparison between the mice fed HFD and ND. (n=6).

Inflammation, especially neuroinflammation, is an important link between obesity and depression. To measure the inflammation in the central nervous system, we examined inflammation markers between HFD mice and normal diet (ND) mice after 8 weeks of high-fat diet (HFD). We found that HFD significantly up-regulated the expression of IL-1β (**Figure 4A**), IL-6 (**Figure 4B**), TNF- α (**Figure 4C**) and NF-κB (**Figure 4D**) in hippocampus.

### BDNF Expression in Hippocampus and Ventromedical Hypothalamus Were Decreased After High Fat Diet

**Figure 5:**
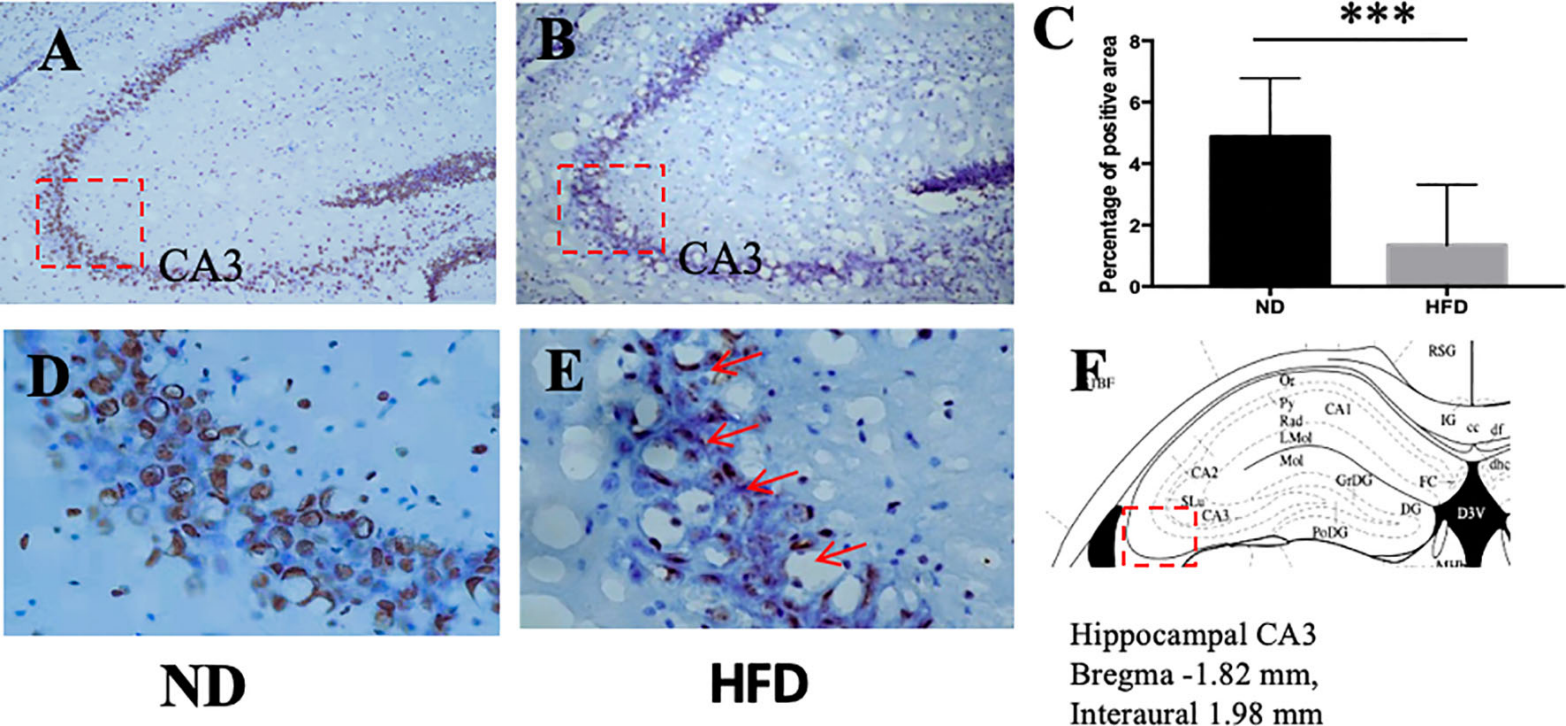
**(A)** BDNF expression in hippocampus in ND group; **(B)** BDNF expression in hippocampus in HFD group; **(C)** percentage of positive area between ND and HFD group; **(D)** higher magnification of **(A)**; **(E)** higher magnification of **(B)**; **(F)** hippocampus area in the brain. ***P value < 0.001, comparison between the mice fed HFD and ND. (n=6).

**Figure 6:**
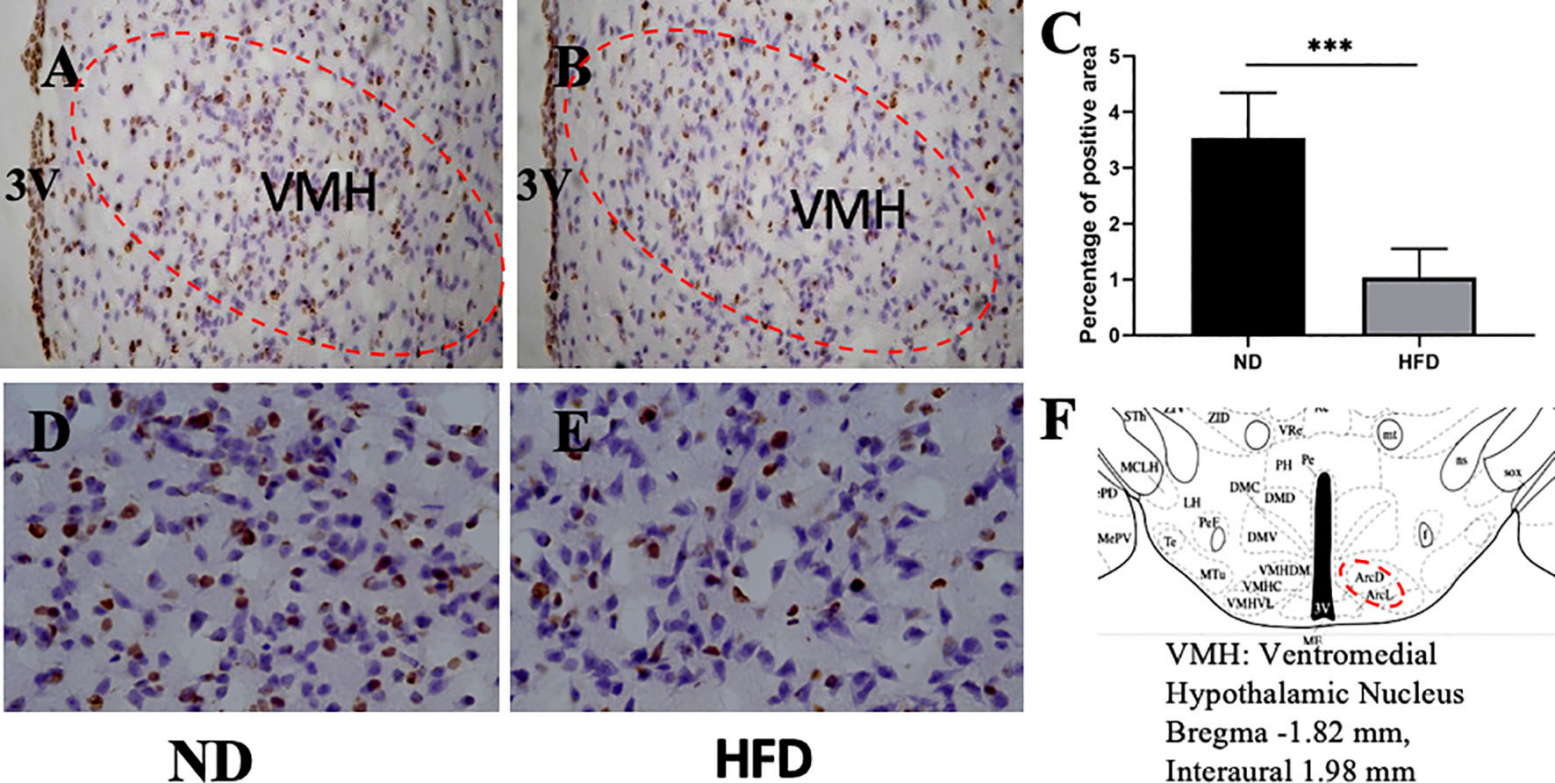
**(A)** BDNF expression in VMH in ND group; **(B)** BDNF expression in VMH in HFD group; **(C)** percentage of positive area between ND group and HFD group; **(D)** higher magnification of **(A)**; **(E)** higher magnification of **(B)**; **(F)** VMH area in the brain. ***P value < 0.001, comparison between the mice fed HFD and ND. (n=6).

BDNF plays a role in emotion regulation, memory function and energy homeostasis as well. To evaluate the role of central BDNF in obesity-related depression, after 8 weeks of high-fat diet (HFD), we examined BDNF expression in hippocampus and ventromedical hypothalamus (VMH). Compared with ND group, BDNF mRNA expression in hippocampus (**Figure 5**), and VMH (**Figure 6**) were significantly down-regulated in HFD group.

### AMPK/CREB Pathway Might Participate in High Fat Diet Induced Depression

**Figure 7:**
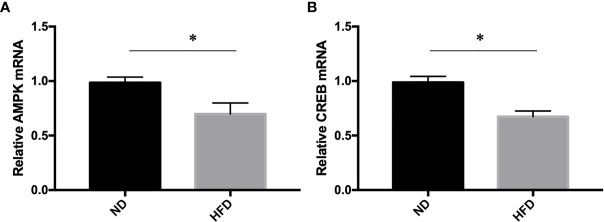
**(A)** AMPK expression; **(B)** CREB expression. *P value < 0.05, comparison between the mice fed HFD and ND. (n=6).

To further understand the molecular events underlying the high-fat diet induced depression-like behavior, q-RT PCR was carried out to measure the expression of AMPK and CREB mRNA expression. Our study showed that AMPK and CREB mRNA expression were decreased in HFD group compared with ND group (**Figure 7**).

## Discussion

The main findings in our present study include (1) high-fat diet can lead to the development of depression through the use of behavioral paradigms; (2) its mechanism is related to the up-regulation of hippocampal GPAT4 expression and hippocampal inflammation; (3) *in situ* hybridization shows BDNF mRNA expression level, down-regulated in hippocampus and VMH; (4) Real-time quantitative PCR detects the down-regulation of hippocampal AMPK and CREB expression levels in the HFD fed mice.

Depression and obesity are closely related, interact, and are supported by a large amount of epidemiological evidence (26). A systematic review and meta-analysis of the longitudinal relationship between depression, overweight, and obesity discovered that obesity increases the risk of depression (27). Vagena et al. (28) discovered that a high-fat diet can promote the development of depression-like behavior in both groups of mice fed with a high-fat diet for 3 weeks and 8 weeks. In this study, we found that a high-fat diet for 8 weeks induces depression-like behavior, which is consistent with previous studies.

However, the specific mechanism of depression caused by obesity needs to be further explored. The hippocampus is a key area that controls emotions and cognitive behavior in the brain. In obese animal models, high levels of hippocampal and cortical cytokines are expressed in this area (29–31).

GPAT catalyze the first step of synthesis of TAG, which also acts as the rate-limiting enzyme in the *de novo* pathway of glycerophospholipid synthesis. Besides, GPAT4 is the only one in the GPAT family that is expressed in the brain including the hippocampus and the cerebellum. The present study reveals that high fat diet induced GPAT4 overexpression in hippocampus, suggesting that GPAT4 in the hippocampus might play a role in diet-induced depression.

Studies have shown that neurotrophins including BDNF have been documented to play a crucial role in depression. BDNF plays a role in emotion regulation and memory function, especially in the hippocampus area (32). The down-regulation of hippocampal BDNF levels is associated with impaired emotion-related behaviors (33). Many kinds of antidepressants and electroconvulsive therapies significantly increase the expression of BDNF in the hippocampus and prefrontal cortex (34). In addition, direct injection of BDNF into the hippocampus can also show antidepressant effects (35). BDNF plays an important regulatory role in the plasticity of hippocampal structure, and mediate protective effects by enhancing neuron survival (36). What’s more, the expression and signal transduction of hippocampal BDNF mRNA negatively regulated by proinflammatory cytokines (37–39). Dexamethasone can reduce the level of pro-inflammatory cytokines, increase the level of anti-inflammatory cytokines, and prevent the decline of BDNF level caused by inflammation (40). The expression of low levels of BDNF in the nervous system may trigger energy homeostasis, thereby developing obesity and glucose intolerance, and metabolic disorders. BDNF is an important part of the central nervous circuit and participates in regulating energy homeostasis (41). Integrate hippocampus BDNF signal affect the efficacy of antidepressants and the anxiety-like behavior (33).

Obviously, BDNF is related to depression. Previous studies found that the expression of BDNF in hippocampus decreased in depressed mice. However, it is not clear how high-fat diet affect BDNF expression in the central nervous system. In this study, we found that HFD simultaneously induced the down-regulation of BDNF mRNA in hippocampus and VMH, suggesting that BDNF may play a role in depression induced by high-fat diet.

VMH is the satiety center in the brain that regulates food intake, glucose and energy metabolism *via* different downstream targets. A recent research discovers the inhibition of peripheral 5-HT synthesis lead to resistance to HFD-induced obesity and can attenuate HFD-induced depression-like behavior (42).VMH is an important center that integrate peripheral metabolic signal (43). Our previous study found that high fat diet-fed mice with impaired glucose tolerance expressed lower level of BDNF mRNA in VMH. HFD leads to changes of BDNF in VMH by affecting the central insulin signaling pathway (44).

Obesity is linked with chronic low-grade inflammation, which actives the peripheral immunity, transform the inflammation in the central nervous system by the humoral, neural and cellular pathways (45). Central inflammation affects the pathophysiological process of depression, including monoaminergic neurotransmission. There were plenty of evidence justify the role of immune inflammatory disorders. A meta-analysis reported that the level of inflammation markers in depressed patients were higher than those in the control group (46–48). For patients with major depression with elevated plasma inflammatory markers, they respond poorly to antidepressant drugs (49). Higher IL-6 and CRP can predict the development of depression (50). Prospective researches also show that depression can predict the later level changes of IL-6 and CRP (51). A meta-analysis including14 randomized placebo controlled trials showed that anti-inflammatory treatments effectively reduce symptoms in patients with depression (52). Higher levels of peripheral IL-6 were related to brain inflammation (53, 54). IL-6 was negatively correlated with hippocampal gray matter volume in healthy adults (53), suggesting that inflammation was a contributing factor to the reduction of hippocampal gray matter. Peripheral inflammation affected hippocampal plasticity, which was due to the activation of microglia and the effects of IL-6 and TNF-α (55, 56). Brain inflammation may negatively affect emotion, study and memory through processes related to neurodegeneration and structural remodeling (57, 58), and mainly affected the hippocampus (59, 60).

5-AMP activated protein kinase (AMPK) is an enzyme involved in energy balance and glucose, and adipose metabolism to help maintain body homeostasis (61, 62). The activation of AMPK can increase the expression of BDNF and active CREB pathway (63). Depression model rats showed overexpression of miR-124 and down-regulation of CREB1 and BDNF in the hippocampus. While knocking down miR-124 improved depression-like behavior in depression rats, which might be related to the increased expression of CREB1 and BDNF in the hippocampus (64). Our study found out downregulation of AMPK and CREB in high-fat fed mice. Depression might be triggered by HFD through AMPK/CREB/BDNF pathway.

There are some limitations in our study. The connection between GPAT4 and BDNF still need to be further confirmed. In our future studies, we will use specific hippocampal GPAT4 knockout mice to further confirm the role of GPAT4 in the hippocampus in the development of depression.

**Figure 8:**
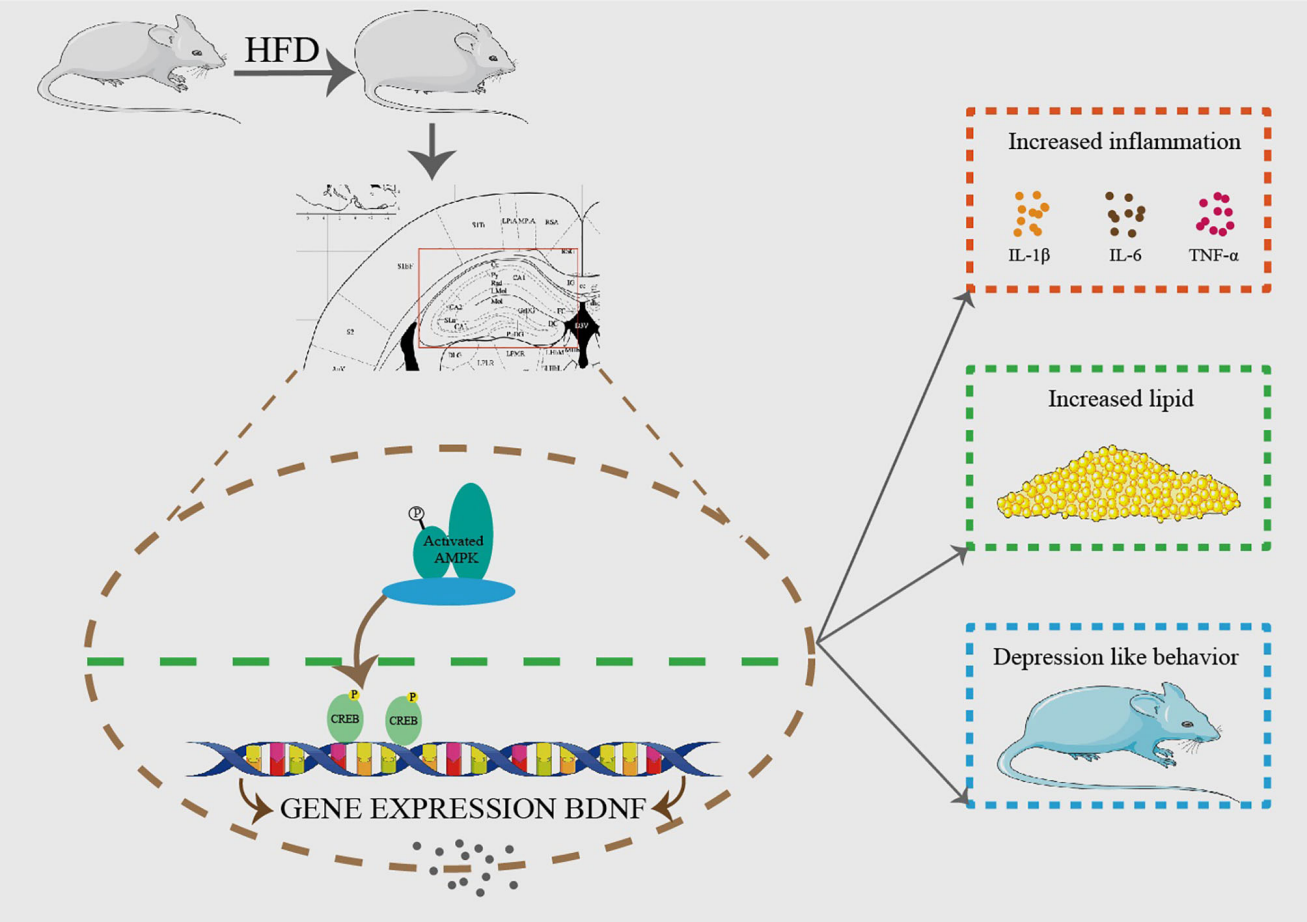
HFD resulted in obesity and depression like behavior. After 8 weeks of HFD, hippocampus GPAT4 and inflammation increased, which was attributed to down-regulation of BDNF, AMP-activated protein kinase (AMPK) and cAMP-response element binding protein (CREB) expression in hippocampus. (n=6).

In conclusion, we demonstrate that hippocampal GPAT4 might participate in HFD induced depression by activating AMPK, CREB and BDNF pathways, which provides insights into a clinical target for obesity-associated depression intervention (**Figure 8**).

## Data Availability Statement

All relevant data is contained within the article. Further inquiries can be directed to Y-qH by yinqiongh@fumu.edu.cn.

## Ethics Statement

The animal study was reviewed and approved by the Experimental Animal Ethics Committee of the Second Affiliated Hospital of Fujian Medical University (2020-388).

## Author Contributions

Y-qH, X-hL and SL conceptualized and designed these studies, performed them, and wrote the manuscript. KH and YW contributed through data analyses, data interpretation, and manuscript preparation. All authors contributed to the article and approved the submitted version.

## Funding

Funding for this work was supported by the Natural science Foundation of Fujian Province (2020J01221); the Key Young Talents Health Training Project of Fujian Province (2020GGA057); Startup Fund for scientific research, Fujian Medical University (2016QH072); Miaopu Fund of the Second Affiliated Hospital of Fujian Medical University (2012MP45).

## Conflict of Interest

The authors declare that the research was conducted in the absence of any commercial or financial relationships that could be construed as a potential conflict of interest.
